# Novel strategy for characterizing the metabolism of GSH–enal adducts in myocardial ischemia rat model using UHPLC–MS

**DOI:** 10.1016/j.jpha.2026.101638

**Published:** 2026-04-17

**Authors:** Jialong Jing, Ning Sheng, Zhe Wang, Jinlan Zhang

**Affiliations:** State Key Laboratory of Bioactive Substance and Function of Natural Medicines, Institute of Materia Medica, Chinese Academy of Medical Sciences & Peking Union Medical College, Beijing, 100050, China

**Keywords:** GSH–Enal adducts, LC-MS, Pre-column derivatization, dMRM, Enal metabolism, Myocardial ischemia

## Abstract

α,β-Unsaturated aldehydes (enals) are primarily metabolized via glutathione (GSH) conjugation, forming GSH–enal adducts, which represent the major pathway for their *in vivo* clearance and detoxification. These adducts undergo further metabolism to form GlyCys–enal adducts, Cys–enal adducts, and N-acetylcysteine (NAC)–enal adducts, ultimately excreted in urine. Elucidating the mechanisms by which enals undergo detoxification through GSH conjugation, metabolism, and excretion is important for understanding their roles in related diseases. However, comprehensive characterization of enal adducts remains challenging because of their structural instability and low concentrations *in vivo*. In this study, a novel strategy was developed to characterize the *in vivo* metabolism of GSH–enal adducts using liquid chromatography-mass spectrometry (LC-MS) combined with 2,4-dinitrophenylhydrazine (DNPH) derivatization. Characteristic fragment ions and fragmentation patterns were summarized based on 56 synthetic enal adducts analyzed by high-resolution MS. Based on the GSH–enal metabolic pathway, a database containing 4496 predicted enal adducts was constructed to facilitate the discovery and identification of potential enal adducts. Using precursor ion scanning by LC-MS/MS integrated with the predicted database and characteristic fragment ions, 499 candidate enal adducts were initially detected in rat heart, liver, and kidney tissues. Among these, 49 novel enal adducts were further identified using dynamic multiple reaction monitoring, and 12 of the identified enal adducts were confirmed using synthetic standards. This strategy was successfully applied to dynamically profile GSH–enal adducts and their metabolites in rats with myocardial ischemia (MI), representing the first comprehensive elucidation of the detoxification and metabolic pathways of oxidative stress-induced enals. These findings provide new insights into the dynamic changes of enals and GSH–enal adducts during disease-associated oxidative damage.

## Highlights

• A novel strategy for comprehensive characterization of GSH–Enal adducts and metabolites.
• For the first time, 49 GSH–Enal adducts and metabolites were identified *in vivo*.
• The profiles of 49 GSH–Enal adducts and metabolites were characterized in MI rat model.

## Graphical abstract

**Figure:**
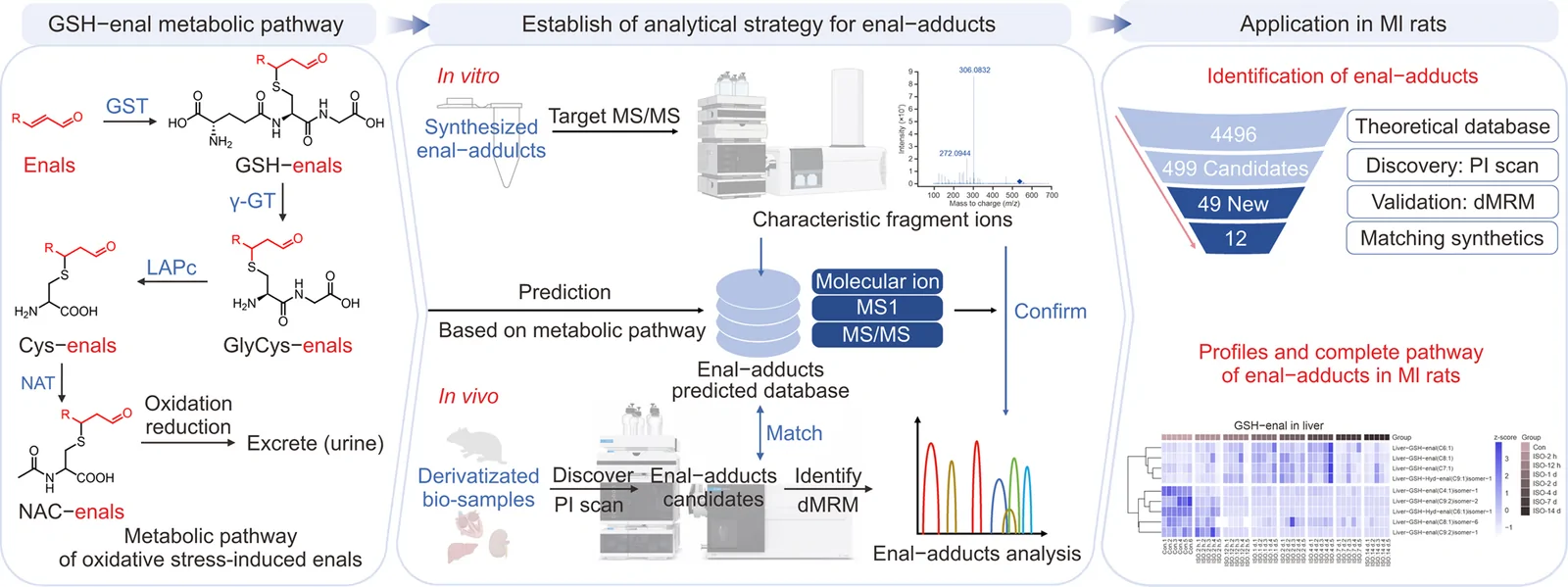

## 1 Introduction

α,β-unsaturated aldehydes, also known as enals, can be ingested exogenously through diet and inhalation, such as during high-temperature cooking of foods or through incomplete combustion of plastics, wood, gasoline, diesel, paraffin, and tobacco [1]. Enals can also be produced endogenously via lipid peroxidation *in vivo* [2]. Owing to their strong electrophilicity and high reactivity, which arise from the conjugation of a carbon–carbon double bond with an aldehyde group, enals readily react with amino acids [3] or nucleotides [4] *in vivo*. These reactions generate enal adducts, including Michael addition products and Schiff bases, which can alter the structure and function of biological macromolecules such as proteins and DNA [5]. As a result, enals pose significant risks to human health [6] and are closely associated with various diseases, particularly cardiovascular diseases [7], neurodegenerative diseases [8], and chronic obstructive pulmonary disease [9]. In our previous study, we developed an “Enalomics” approach [10], which demonstrated that myocardial injury is associated with disordered enal metabolism and that plasma enals can serve as potential biomarkers for the diagnosis of myocardial ischemia (MI). The present study focuses on the subsequent metabolism of these enals through conjugation with glutathione (GSH) in tissues from normal and MI model rats, with particular emphasis on the *in vivo* detoxification pathways of enals. This work represents an in-depth extension of our previous research and provides analytical methods and strategies for investigating oxidative stress homeostasis imbalance and antioxidant drug targets.

Conjugation with GSH constitutes the principal metabolic and elimination pathway for enals *in vivo* in humans [2]. GSH, a crucial endogenous antioxidant [11,12], is a tripeptide composed of glutamic acid (Glu), cysteine (Cys), and glycine (Gly). In addition to scavenging free radicals, maintaining intracellular redox homeostasis, and protecting cells from oxidative damage, GSH plays a central role in detoxification by facilitating the elimination of harmful compounds [13]. As illustrated in Fig. 1, the *in vivo* metabolic pathway of GSH–enal adducts proceeds as follows: enals generated by fatty acid peroxidation in the heart undergo a Michael addition reaction between the thiol group of GSH and the carbon–carbon double bond of enals, catalyzed by glutathione S-transferase (GST) in the liver [14,15], to form GSH–enal adducts. Subsequently, γ-glutamyltransferase (γ-GT) [16] catalyzes cleavage of the γ-glutamyl bond, resulting in the removal of Glu and formation of GlyCys–enal adducts. Further hydrolysis by the GlyCys peptide bond by leucyl aminopeptidase (LAPc) [17] releases Gly, yielding Cys–enal adducts. These Cys–enal adducts are transported via the bloodstream to the kidneys, where N-acetylation of Cys is catalyzed by N-acetyltransferase (NAT) [18] to form N-acetylcysteine–enal conjugates (NAC–enals). Finally, the aldehyde group of enals undergoes redox reactions [2], producing hydroxylated or carboxylated metabolites that are excreted in urine [19,20]. Collectively, these GSH–enal adducts and their downstream metabolites (hereafter referred to as enal adducts) are closely associated with the detoxification, metabolism, and excretion of enals *in vivo*. Elucidating the mechanism by which enals undergo detoxification through GSH conjugation, metabolism, and excretion is essential for understanding oxidative stress-related diseases.

**Fig. 1:**
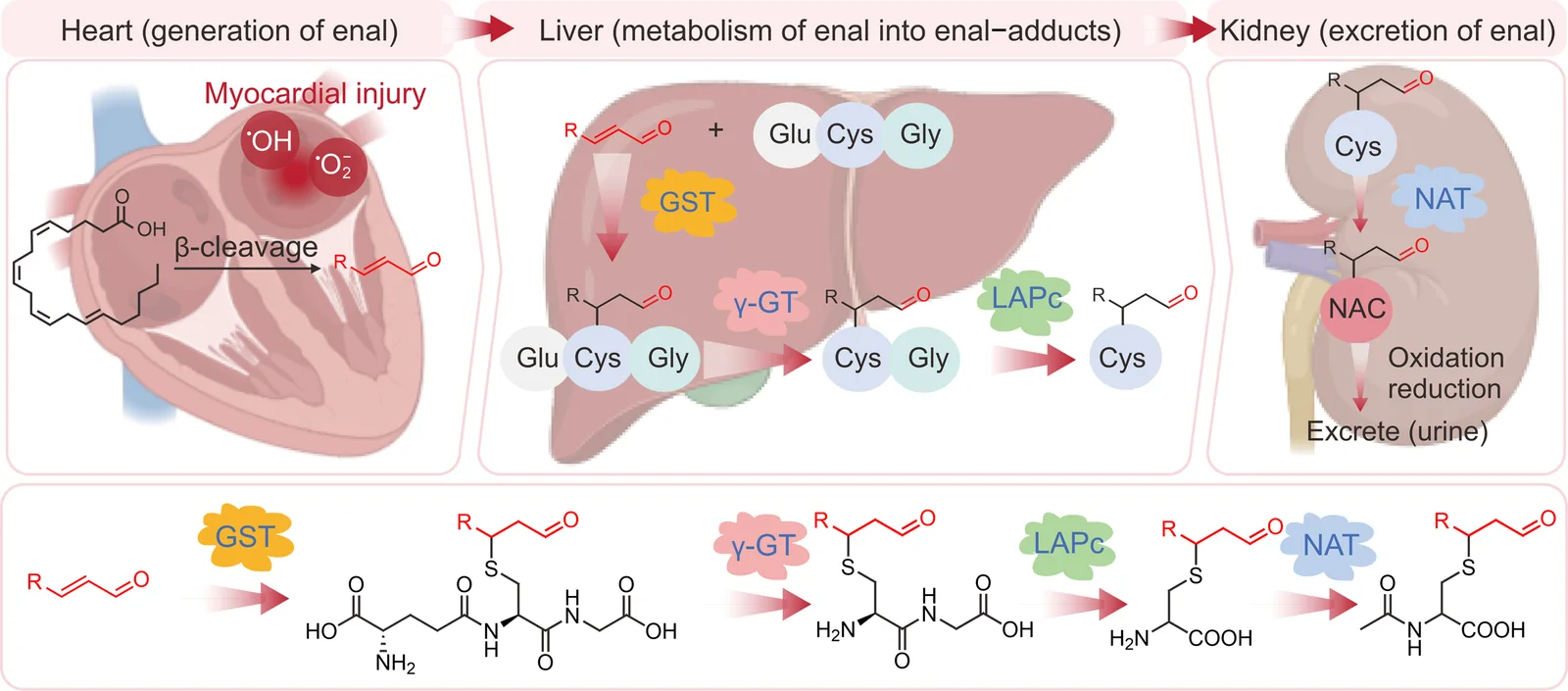
Glutathione (GSH)–enal metabolic pathway *in vivo*: enals are produced in the heart, undergo adduct metabolism in the liver, and are excreted in the kidneys (top) and structure of enal adducts during enal metabolism (bottom). Glu: glutamic acid; Cys: cysteine; Gly: glycine; NAC: N-acetylcysteine; GST: glutathione S-transferase; γ-GT: γ-glutamyltransferase; LAPc: leucyl aminopeptidase; NAT: N-acetyltransferase.

Owing to the low abundance of enal adducts *in vivo*, limited detection sensitivity resulting from poor ionization efficiency, structural instability, and the lack of authentic standards, the analysis of enal adducts and their complete *in vivo* metabolic pathways remains highly challenging. To date, there are very few *in vivo* studies of enal adducts. Most reported studies have relied on chemical synthesis *in vitro* [21] or cellular exposure to enals [22,23] to generate GSH–enal adducts or NAC–enals, which are subsequently identified by liquid chromatography-mass spectrometry (LC-MS). However, the *in vivo* environment is far more complex than *in vitro* systems, and these approaches are insufficient to fully elucidate the metabolic pathways and exposure profiles of enal adducts *in vivo*. Therefore, there is an urgent need to develop a novel analytical strategy for the comprehensive characterization and identification of GSH–enal adducts and their metabolites *in vivo*.

In this study, we developed a novel analytical strategy for the comprehensive characterization of GSH–enal adducts and their metabolites *in vivo*, integrating knowledge of enal metabolic pathways with derivatization-assisted LC-MS analysis. Derivatization with 2,4-dinitrophenylhydrazine (DNPH) markedly improved the ionization efficiency and chromatographic performance of enal adducts, thereby enhancing detection sensitivity. In addition, a predicted database of potential enal adducts was constructed based on known metabolic pathways to facilitate their discovery and identification *in vivo*. Characteristic fragment ions and fragmentation patterns of *in vitro*-synthesized enal adducts were systematically summarized using ultra-high-performance liquid chromatography-quadrupole time-of-flight mass spectrometry (UHPLC-Q-TOF/MS). Precursor ion scanning with ultra high performance liquid chromatography triple quadrupole mass spectrometry (UHPLC-QqQ/MS), using these characteristic fragments as product ions, was first employed for the preliminary screening of enal adducts *in vivo*. The candidate enal adducts identified were further validated by dynamic multiple reaction monitoring (dMRM), using their quasi-molecular ions as precursor ions and additional characteristic fragments as product ions. Finally, this strategy was applied to a rat model of MI, revealing temporal variation profiles of enal products with different structural features following MI. This work provides robust technical support and new insights for the comprehensive characterization of endogenous enal adducts and for in-depth investigations into their associations with disease.

## 2 Materials and methods

### 2.1 Chemicals and materials

Enal standards (Fig. S1) were purchased from J&K Scientific Ltd. (Beijing, China) and Zesheng Technology Co., Ltd. (Anhui, China). DNPH and NAC were obtained from Aladdin (Shanghai, China). GSH and Cys were purchased from J&K Scientific Ltd. (Beijing, China), while GlyCys was obtained from Acmec Biochemical Technology Co., Ltd. (Shanghai, China). Ascorbic acid (AA) was purchased from J&K Scientific Ltd. (Beijing, China). Isoprenaline hydrochloride (99%) was obtained from MedChemExpress (Shanghai, China). Detailed information on other chemicals and reagents is shown in the Supplementary data.

### 2.2 *In vitro* synthesis and sample derivatization

The *in vitro* synthesis of enal adducts is described in the Supplementary data. For derivatization, 100 μL of a 5 mmol/L DNPH solution was added to the prepared GSH–enal adducts, vortex-mixed thoroughly, and incubated in a 37 °C water bath in the dark for 1 h to obtain the derivatized products. The preparation procedures for GlyCys–enal–DNPH, Cys–enal–DNPH, and NAC–enal–DNPH were identical to that used for GSH–enal–DNPH.

### 2.3 Biological sample pretreatment

An aliquot of 100 μL of rat tissue homogenate (heart, liver, or kidney) was accurately transferred into a tube, and 100 μL of a 5 mmol/L DNPH solution was added. The mixture was vortex-mixed and incubated in a shaking water bath at 37 °C for 1 h. After the reaction, 1 mL of methanol/methyl tert-butyl ether (MTBE) (1:1, v/v) was added to precipitate proteins. The mixture was vortexed for 10 min and centrifuged at 12,000 rpm for 10 min. The supernatant was collected, and the solvent was evaporated to dryness under vacuum. The residue was reconstituted in 50 μL of acetonitrile/water (1:1, v/v) prior to analysis.

### 2.4 UHPLC-Q-TOF/MS analysis

*In vitro*-synthesized enal adducts were analyzed using an Agilent 1290 Series UHPLC system coupled to an Agilent 6550 Q-TOF mass spectrometer (Agilent Technologies, Santa Clara, CA, USA). Chromatographic separation was performed on a Waters ACQUITY UPLC BEH C18 column (2.1 mm × 100 mm, 1.7 μm; Milford, MA, USA) using an 18 min gradient elution. The mass spectrometer was operated in negative electrospray ionization (ESI) target MS/MS mode. Detailed target MS/MS parameters are provided in Tables S1–S4, and additional experimental conditions are described in the Supplementary data.

### 2.5 UHPLC-QqQ/MS analysis

Analysis of enal adducts in biological samples was performed using an Agilent 1290 Series UHPLC system coupled to an Agilent 6470 triple quadrupole mass spectrometer (Agilent Technologies, Santa Clara, CA, USA). Chromatographic separation was performed on a Waters ACQUITY UPLC BEH C18 column (2.1 mm × 100 mm, 1.7 μm; Milford, MA, USA) using a 45 min gradient elution. Detailed chromatographic and mass spectrometric conditions are provided in the Supplementary data.

Precursor ion scanning mode was employed to discover endogenous GSH–enal, GlyCys–enal, Cys–enal, and NAC–enal adducts. The diagnostic product ions were set to *m/z* 306.1 for GSH–enal–DNPH, *m/z* 177.0 for GlyCys–enal–DNPH, *m/z* 120.0 for Cys–enal–DNPH, and *m/z* 162.0 for NAC–enal–DNPH.

dMRM mode was subsequently applied to validate endogenous GSH–enal, GlyCys–enal, Cys–enal, and NAC–enal adducts. The confirmatory product ions were *m/z* 272.1 for GSH–enal–DNPH, [M−177−2H]^-^ for GlyCys–enal–DNPH, [M−120−2H]^-^ for Cys–enal–DNPH, and [M−162−2H]^-^ for NAC–enal–DNPH.

To comprehensively characterize alterations in the complete *in vivo* enal metabolic pathway, parent enals were also analyzed in addition to enal adducts. The analytical method for enal detection was adopted from our previous work [10].

### 2.6 Identification of enal adducts

First, a theoretically predicted database of enal adducts was established based on the chemical structures and *in vivo* metabolic pathways of enals. Characteristic fragment ions and fragmentation patterns of *in vitro*-synthesized enal adducts were systematically summarized using UHPLC-Q-TOF/MS. Using these characteristic fragments as diagnostic product ions in combination with the predicted database, precursor ion scanning was performed with UHPLC-QqQ/MS to preliminarily screen candidate enal adducts. The quasi-molecular ions of the candidate enal adducts were then selected as precursor ions, and additional characteristic fragments were used as product ions to validate the identified enal adducts and their metabolites *in vivo* by dMRM using UHPLC-QqQ/MS.

### 2.7 Animal experiments

Male Sprague-Dawley (SD) rats (250 ± 20 g, purchased from SPF Biotechnology Co., Ltd., Beijing, China) were randomly assigned to two groups: control (Con, *n* = 6) and isoproterenol induced myocardial ischemia (ISO, *n* = 35). The ISO group was further subdivided into seven subgroups based on sampling time points: ISO-2 h, ISO-12 h, ISO-1 d, ISO-2 d, ISO-4 d, ISO-7 d, and ISO-14 d (*n* = 5 per subgroup). Myocardial injury was induced by isoproterenol (5 mg/kg/day) as previously described [24]. Heart, liver, and kidney tissues were supplemented with 0.5% AA [10] during homogenization to prevent *ex vivo* oxidation. Tissues were collected for analysis of enal adducts, hematoxylin and eosin (H&E) staining of the heart, and biochemical assays. All animal protocols were approved by the Institutional Animal Care and Welfare Committee of the Chinese Academy of Medical Sciences & Peking Union Medical College (Approval No.: 00002254; Approval date: September 5, 2024). Detailed experimental procedures are provided in the Supplementary data.

### 2.8 Data analysis

Data were analyzed using MassHunter Qualitative Analysis software (version B.10.0, Agilent Technologies, Santa Clara, CA, USA). Comparison between groups was performed using analysis of variance (ANOVA) with Brown-Forsythe and Bartlett tests. A *P*-value < 0.05 was considered statistically significant. Boxplots and line graphs were generated using GraphPad Prism 8.0.2 (GraphPad Software, San Diego, CA, USA). Metabolite concentrations in tissue samples were normalized to protein content. The theoretically predicted database of enal adducts was created using Microsoft Excel. Heatmaps were generated in R. Some graphical elements were obtained from SciDraw (www.scidraw.io).

## 3 Results and discussion

### 3.1 Development and optimization of analytical methods for enal adducts

Due to the lack of commercial standards for enal adducts, developing analytical methods based on their chromatographic and mass spectrometric properties (e.g., retention time, characteristic fragment ions) is challenging, which has hindered the discovery and identification of enal adducts *in vivo*. As illustrated in Fig. 2A, this limitation was addressed by synthesizing, for the first time, four categories comprising a total of 56 enal adducts *in vitro*. Their structures are detailed in Figs. S2–S5. However, direct detection by ESI-MS exhibited poor sensitivity due to the high polarity and low ionization efficiency of these compounds. Moreover, structural similarity among enal adducts within the same category led to incomplete chromatographic separation and co-elution interference. To overcome these challenges, derivatization with DNPH was implemented (Fig. 2A), following established protocols for *in vivo* enal analysis [10]. Derivatization efficiency was systematically optimized by varying reaction temperature and duration (Fig. 2B), leading to the final conditions: incubation in a 37 °C water bath for 1 h, which achieved maximal derivatization yield.

**Fig. 2:**
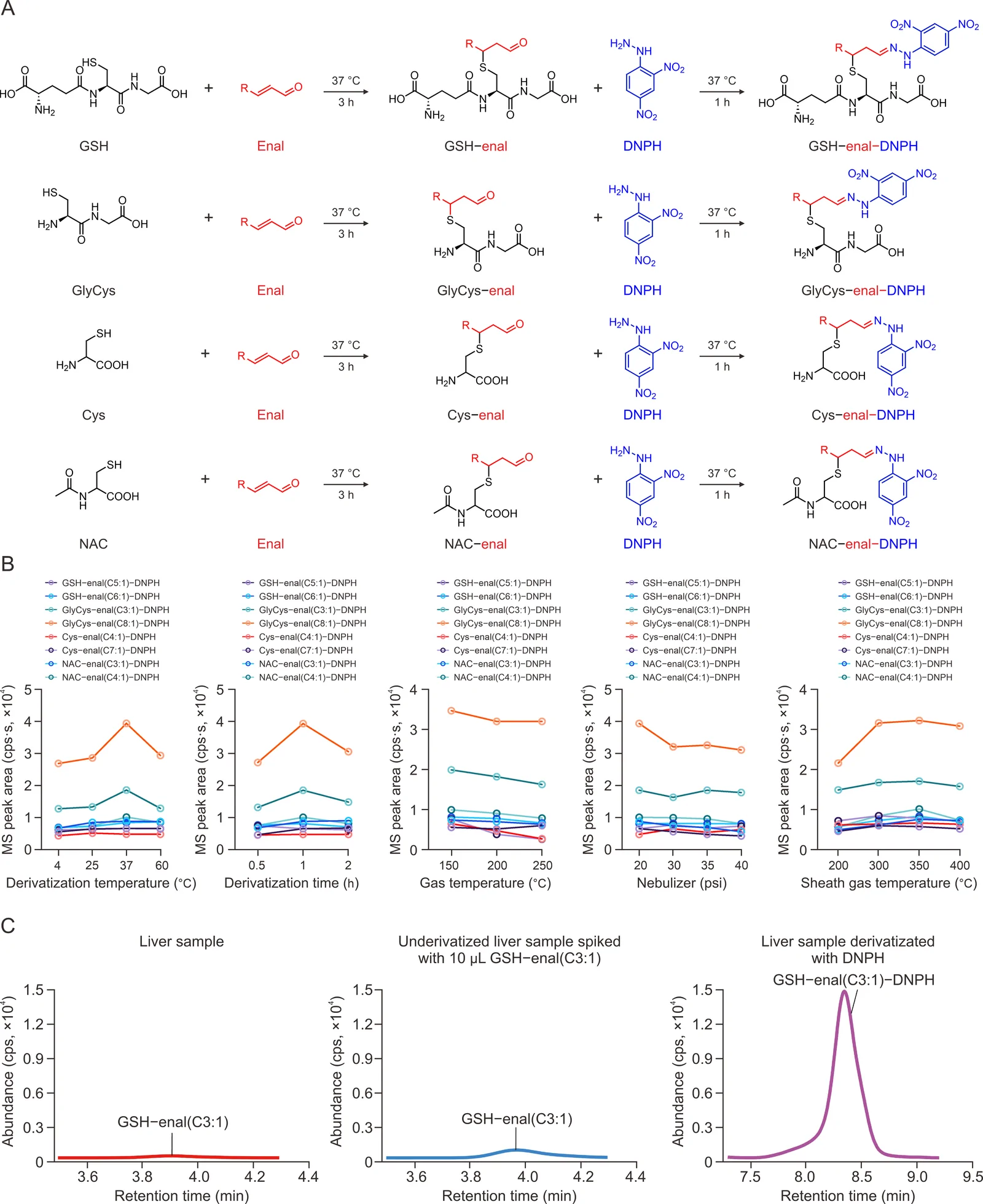
Establishment and optimization of analytical methods for enal adducts. (A) Structure and synthetic pathway of 2,4-dinitrophenylhydrazine (DNPH)-derivatized enal adducts. (B) Optimization of derivatization conditions and mass spectrometry (MS) parameters. (C) Mass spectral response of underivatized liver sample, underivatized liver sample spiked with 10 μL glutathione (GSH)–enal(C3:1), and liver sample after DNPH derivatization. NAC: N-acetylcysteine.

The efficacy of DNPH derivatization was evaluated by comparing MS signal intensities in the three sample groups: (1) underivatized rat liver homogenate, (2) rat liver homogenate spiked with underivatized GSH–enal(C3:1), and (3) rat liver homogenate subjected to DNPH derivatization. As shown in Fig. 2C, the endogenous enal adduct signal intensity in untreated liver samples was 2.75 × 10^2^. Spiking with 10 μL of GSH–enal(C3:1) reaction mixture increased the non-derivatized signal to 1 × 10^3^, whereas DNPH derivatization enhanced the signal to 1.60 × 10^4^, representing an approximately 60-fold improvement in MS response. These results demonstrate that DNPH–mediated derivatization significantly improves the detection sensitivity of enal adducts.

To predict chromatographic retention behavior on a C18 column, the octanol-water partition coefficient (Log *P*) was calculated [25]. Enal(C3:1) adducts, being the shortest–chain adducts, exhibit the lowest Log *P* value among all enal adducts. If their DNPH–derivatized products show sufficient retention on a C18 column, longer-chain enal-adduct derivatives are expected to exhibit even greater retention due to higher hydrophobicity. Computational analysis indicated that non-derivatized GSH–enal(C3:1), GlyCys–enal(C3:1), Cys–enal(C3:1), and NAC–enal(C3:1) have Log *P* values of −3.68, −2.52, −1.37, and −1.44, respectively. After DNPH derivatization, these values increased to −2.18 (GSH–enal–DNPH), −0.75 (GlyCys–enal–DNPH), 0.58 (Cys–enal–DNPH), and 0.91 (NAC–enal–DNPH). The substantial increase in Log P indicates that DNPH derivatization reduces molecular polarity and enhances efficiency on the C18 column.

To enhance detection sensitivity, ion source parameters were systematically optimized by evaluating the peak areas of DNPH-derivatized enal adducts under varying conditions: drying gas temperature (150 °C, 200 °C, 250 °C), nebulizer voltage (20 psi, 30 psi, 35 psi, 40 psi), and sheath gas temperature (200 °C, 300 °C, 350 °C, 400 °C). As shown in Fig. 2B, the optimal settings were determined to be 150 °C for the drying gas, 20 psi for the nebulizer voltage, and 350 °C for the sheath gas. Additionally, collision energy (CE) was optimized by comparing fragment ion abundance at 10 eV, 20 eV, and 30 eV. Based on these results (Fig. S6), a CE of 20 eV was selected for subsequent analyses, providing improved signal stability and fragmentation efficiency.

Extraction efficiency of DNPH-derivatized enal adducts from biological samples was evaluated using eight solvent systems: acetonitrile, methanol, acetonitrile with 0.2% formic acid, methanol with 0.2% formic acid, ethyl acetate, n-hexane, MTBE–methanol (1:1, v/v), and MTBE–acetonitrile (1:1, v/v). As shown in Fig. S7, MTBE–methanol (1:1, v/v) exhibited significantly higher recovery rates compared to other solvents, indicating its superior suitability for extracting these polar analytes from complex biological matrices.

Finally, four analytical methods were established to quantify GSH–enal, GlyCys–enal, Cys–enal, and NAC–enal adducts in biological samples. Method validation was performed using 12 synthetic enal adducts in accordance with International Council for Harmonisation (ICH) M10 guidelines [26], with results summarized in Tables S5 and S6. The limits of detection for the 12 enal adducts ranged from 1 to 200 μmol/L, and the linear correlation coefficients (*r*^2^) were all greater than 0.99. Accuracy ranged from 81.6% to 115%, and the intra- and inter-day precision relative standard deviation (RSD) ranged from 1.7% to 15%. The matrix effect ranged from 86.7% to 120% with RSDs ≤14%, and extraction recovery was stable with RSDs ≤ 9.1%.

### 3.2 Establishment of a theoretical predicted database for *in vivo* enal adducts

To validate the DNPH-derivatized enal adducts synthesized *in vitro* and identify their characteristic fragment ions, the established HPLC-Q-TOF/MS method was applied to analyze the synthetic samples. Results are presented in Figs. S8–S11. GSH–enal–DNPH, GlyCys–enal–DNPH, Cys–enal–DNPH, and NAC–enal–DNPH consistently yielded two types of characteristic fragments: Type I fragments: Resulting from cleavage of the adduct to generate GSH-, GlyCys-, Cys-, or NAC-containing moieties, with corresponding *m/z* values of 306.1, 177.0, 120.0, and 162.0, respectively. Type II fragments: Corresponding to the DNPH-derivatized enal moiety, serving as a signature for enal identification. This dual-fragmentation pattern confirms the structural integrity of both the thiol-conjugated and DNPH-derivatized portions of the adducts.

Using enal(C5:1) as a model compound (Fig. 3A), high-resolution MS (HRMS) confirmed the successful synthesis and DNPH derivatization of all four enal-adduct classes. The precursor ion of GSH–enal(C5:1)–DNPH was observed at *m/z* 570.1683, corresponding to the calculated molecular formula C_21_H_29_N_7_O_10_S (theoretical *m/z* 570.1624; error: +10.3 ppm), with two characteristic fragment ions at *m/z* 272.0948 and 306.0833, representing GSH-derived structural motifs. Similarly, GlyCys–enal(C5:1)–DNPH exhibited a precursor ion at *m/z* 441.1248 (C_16_H_22_N_6_O_7_S; theoretical *m/z* 441.1198; error +11.3 ppm) and fragment ions at *m/z* 177.0389 (GlyCys-specific) and 263.0845 (enal–DNPH-specific). Cys–enal(C5:1)–DNPH showed a precursor ion at *m/z* 384.0984 (C_14_H_19_N_5_O_6_S; theoretical *m/z* 384.0983; error: +0.2 ppm), with fragment ions at *m/z* 120.0126 (Cys-specific) and 263.0795 (enal–DNPH-specific). NAC–enal(C5:1)–DNPH displayed a precursor ion at *m/z* 426.1068 (C_16_H_21_N_5_O_7_S; theoretical *m/z* 426.1089; error −4.9 ppm), producing fragment ions at *m/z* 162.0215 (NAC-specific) and 263.0769 (enal–DNPH-specific). The consistent detection of these characteristic fragments, including thiol-conjugate-derived and enal–DNPH-derived ions, validates the structural integrity of all four synthesized adduct classes, confirming their utility for unambiguous identification of enal adducts in *in vivo* studies. A summary of characteristic fragment ions and the structures of representative enal(C5:1) adducts is provided in Table 1, with their mass spectrometric fragmentation rules illustrated in .Figs. S12–S15

**Fig. 3:**
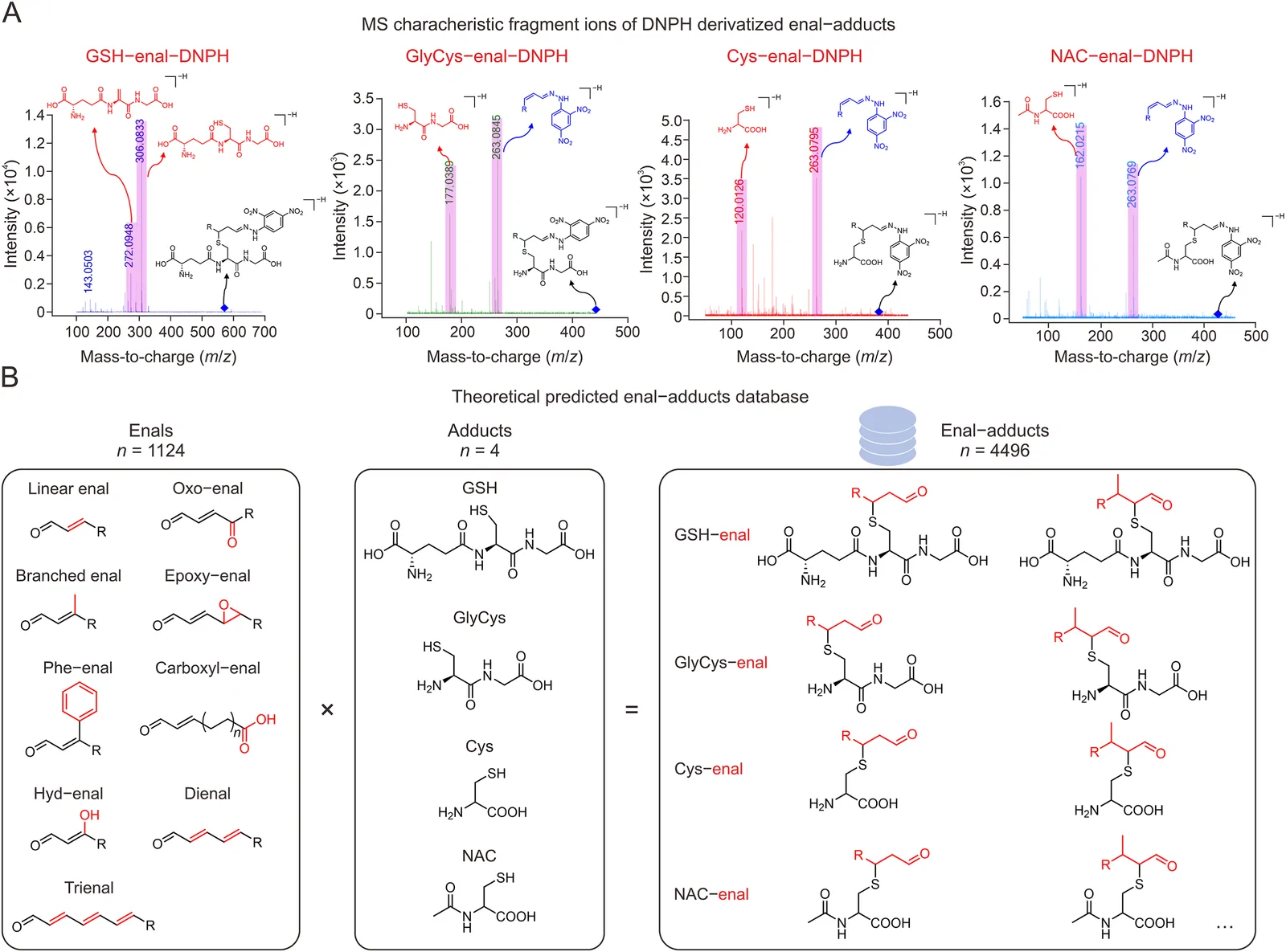
Establishment of the theoretical predicted database of enal adducts. (A) Mass spectrometry (MS) characteristic fragment ions of 2,4-dinitrophenylhydrazine (DNPH)-derivatized enal adducts. (B) Theoretical predicted enal–adduct database. GSH: glutathione; Cys: cysteine; Gly: glycine; NAC: N-acetylcysteine.

**Table 1:** Summary of characteristic fragment ions and structures of representative enal−adducts.

Compound	Formula	[M−H]^–^ (*m/z*)	Characteristic fragment ion–1 (*m/z*)	Characteristic fragment ion-1 mechanisms	Characteristic fragment ion-1 structure	Characteristic fragment ion-2 (*m/z*)	Characteristic fragment ion-2 mechanisms	Characteristic fragment ion-2 structure
GSH–enal(C5:1)–DNPH	C_21_H_29_N_7_O_10_S	570.1683	272.0948	GSH–specific (cleavage of the C–S bond in GSH–enal–DNPH)		306.0833	GSH–specific (cleavage of the C–S bond in GSH–enal–DNPH)	
GlyCys–enal(C5:1)–DNPH	C_16_H_22_N_6_O_7_S	441.1248	177.0389	GlyCys–specific (cleavage of the C–S bond in GlyCys–enal–DNPH)		263.0845	enal–DNPH–specific (cleavage of the C–S bond in GlyCys–enal–DNPH)	
Cys–enal(C5:1)–DNPH	C_14_H_19_N_5_O_6_S	384.0984	120.0126	Cys–specific (cleavage of the C–S bond in Cys–enal–DNPH)		263.0795	enal–DNPH–specific (cleavage of the C–S bond in Cys–enal–DNPH)	
NAC–enal(C5:1)–DNPH	C_16_H_21_N_5_O_7_S	426.1068	162.0215	NAC–specific (cleavage of the C–S bond in NAC–enal–DNPH)		263.0769	enal–DNPH–specific (cleavage of the C–S bond in NAC–enal–DNPH)	

GSH: glutathione; Gly: glycine; Cys: cysteine; NAC: N-acetylcysteine; DNPH: 2,4-dinitrophenylhydrazine.

Building on this foundation, a theoretically predicted database of *in vivo* enal adducts was established. As illustrated in Fig. 3B, to ensure comprehensive coverage of enal adducts across different structural types, we first compiled 1124 endogenous enals with carbon chain lengths ranging from C3 to C28, based on our previous study and the Human Metabolome Database (HMDB) database (Table S7, [10]). These enals were classified into nine structural classes: linear enal, branched enal, Phe–enal, Hyd–enal, oxo–enal, epoxy–enal, carboxyl–enal, dienal, and trienal. Next, combinatorial pairing of these enals with four thiol-containing partners (GSH, GlyCys, Cys, NAC) generated a database encompassing 4496 theoretically predicted enal adducts, corresponding to the four major thiol-derived categories. As detailed in Table S8, the database includes information on compound names, molecular formulas, exact molecular weights, predicted HRMS data and characteristic fragment ions. This predicted database provides a robust tool for the comprehensive discovery and validation of endogenous enal adducts *in vivo*.

### 3.3 Discovery and identification of endogenous enal adducts

As illustrated in Fig. 4A, an analytical strategy was established for the comprehensive discovery and identification of enal adducts *in vivo*. Representative enal adducts synthesized *in vitro* were first derivatized with DNPH, and their characteristic fragment ions were identified via UHPLC-Q-TOF/MS analysis. Based on enal structures and metabolic pathways, a theoretical predicted database containing 4496 enal-adduct candidates was constructed. Using the type I fragment ions as product ions, precursor ion scanning with UHPLC-QqQ/MS was performed and matched against the predicted database, enabling the discovery of potential enal adducts in rat heart, liver, and kidney tissues. Subsequently, these candidates were validated through dMRM scanning on UHPLC-QqQ/MS, targeting the type II fragment ions as daughter ions. This integrated workflow ensures both broad coverage and high-confidence characterization of enal adducts *in vivo*.

**Fig. 4:**
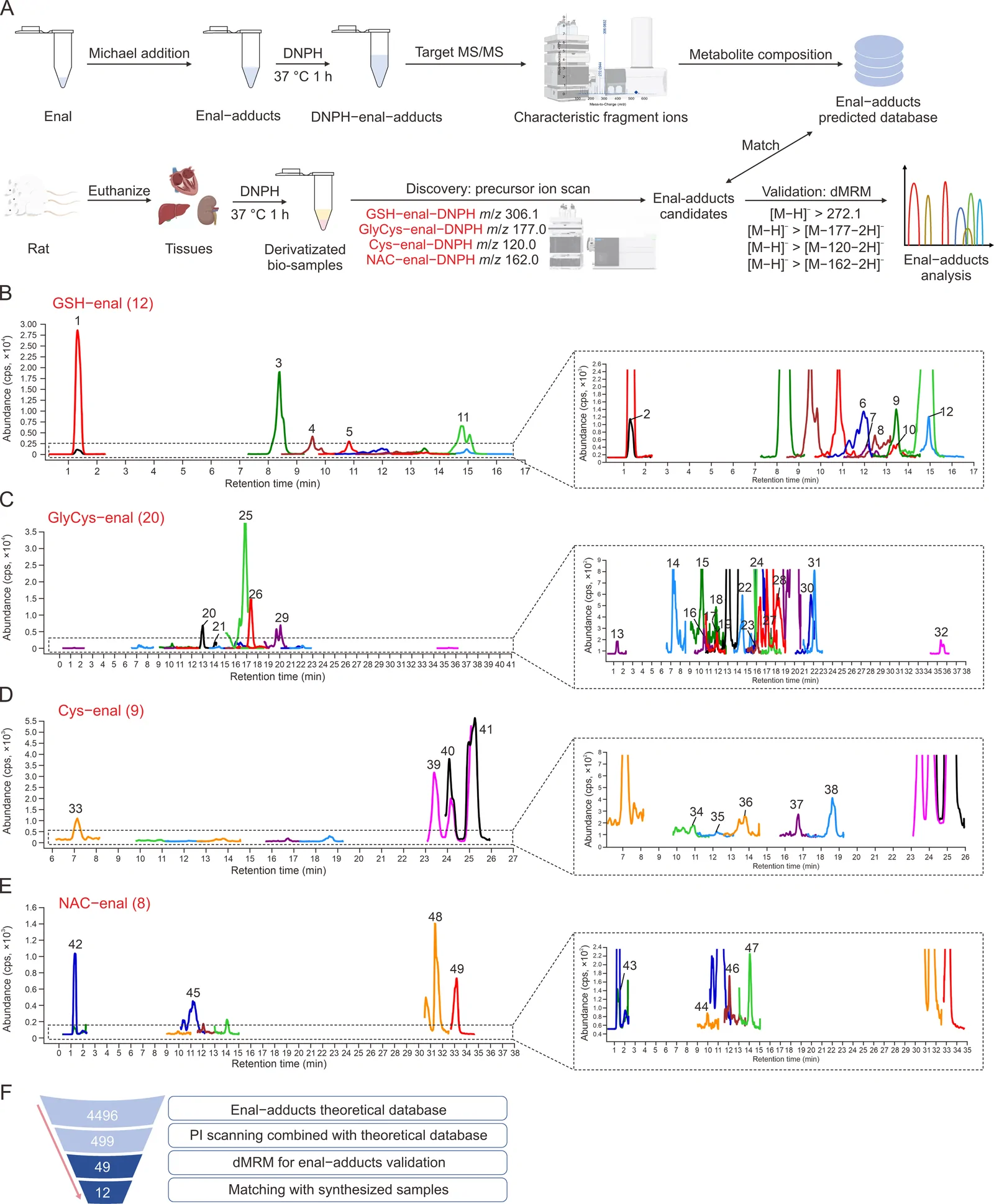
Enal–adduct profiling in rat tissues. (A) Flowchart of the *in vivo* enal–adduct analytical strategy. (B–E) Overlapped multiple reaction monitoring (MRM) chromatograms of identified enal adducts in rat tissues: glutathione (GSH)–enals (B), GlyCys–enals (C), Cys–enals (D), and N-acetylcysteine (NAC)–enals (E). Enal adducts in panels B–E are numbered in Table S13. (F) Summary of enal–adducts in rat tissues characterized by liquid chromatography-mass spectrometry (LC-MS). DNPH: 2,4-dinitrophenylhydrazine; dMRM: dynamic multiple reaction monitoring; PI: precursor ion.

Following this strategy, rat heart, liver, and kidney tissues were homogenized, extracted, and derivatized. Precursor ion scanning was conducted using GSH-derived fragment ion (*m/z* 306.1), GlyCys-derived fragment ion (*m/z* 177.0), Cys-derived fragment ion (*m/z* 120.0), and NAC-derived fragment ion (*m/z* 162.0) as product ions. As shown in Fig. S16, 74 GSH–enal, 277 GlyCys–enal, 74 Cys–enal, and 74 NAC–enal candidates were initially identified (Tables S9–S12). For validation, dMRM scans with higher sensitivity were performed: For GSH–enal candidates, precursor ions of DNPH-derivatized adducts were paired with the GSH fragment ion (*m/z* 272.1) as the product ion (Fig. 4B), validating 12 GSH–enal adducts. For GlyCys–enal candidates, precursor ions were paired with the enal–DNPH fragment ion (*m/z* [M−177−2H]^-^), validating 20 GlyCys–enal adducts (Fig. 4C). For Cys–enal candidates, precursor ions were paired with the enal–DNPH fragment ion (*m/z* [M−120−2H]^-^), validating 9 Cys–enal adducts (Fig. 4D). For NAC–enal candidates, precursor ions were paired with the enal–DNPH fragment ion (*m/z* [M−162−2H]^-^), identifying 8 NAC–enal adducts (Fig. 4E). All verified enal adducts are summarized in Table S13, which includes compound names, types, structures, retention times (min), precursor and product ions (*m/z*), collision energies (eV), polarity, and the other organs in which they were detected. These represent the first *in vivo* identification of these compounds, with no prior literature documenting their occurrence *in vivo*.

As shown in Fig. 4F, the workflow reduced 4496 theoretical predicted enal-adduct candidates to 499 potential matches via precursor ion scanning and database alignment. Subsequent dMRM validation confirmed 49 enal adducts, with 12 matching synthetically prepared *in vitro* references, satisfying proposed minimum reporting standards for chemical analysis [27]. By integrating *in vitro* synthesis of references, DNPH derivatization (to enhance ionization efficiency and sensitivity), and a workflow combining database-driven precursor ion scanning with pseudo-targeted dMRM analysis, the method systematically reduced candidate compounds, eliminating 80%–90% of potential interfering ions. This approach enabled comprehensive discovery and accurate identification of the four major enal–adduct classes *in vivo*, addressing key challenges such as the lack of commercial standards and low *in vivo* detection sensitivity.

### 3.4 Dynamic changes of enal(C3:1), enal(C4:1), enal(C5:1), and their adducts across multiple tissues in myocardial ischemic rats

Because human heart, liver, and kidney samples from MI cases are difficult to obtain, the SD rat MI model was used as a reference to investigate *in vivo* detoxification and metabolic processes of enals. A MI rat model was established via intraperitoneal injection of isoproterenol, as detailed in Fig. S17. Model validation was confirmed by histopathological analysis of cardiac sections using H&E staining (Fig. S18), measurement of plasma aspartate aminotransferase (AST) levels, and assessment of heart superoxide dismutase (SOD) activity (Fig. S19). Based on H&E staining results (Fig. S18) and literature on post-MI recovery phases [28,29], the acute inflammatory phase was defined as 2 h to 2 days post-ISO administration, whereas the tissue reparative and proliferative phase spanned 4 days to 14 days post-injury.

Oxidative stress [30], characterized by excessive production of reactive oxygen species (ROS) that induce molecular damage, is a hallmark of myocardial ischemic injury [31]. Enals, a class of reactive aldehydes, are generated *in vivo* through ROS-mediated lipid peroxidation of unsaturated fatty acids [32]. These enals undergo detoxification via conjugation with GSH to form GSH–enal adducts, which are further metabolized into GlyCys–enal, Cys–enal, and NAC–enal adducts, representing a critical pathway for enal elimination *in vivo*. In this study, the four major enal–adduct classes derived from enal(C3:1), enal(C4:1), and enal(C5:1) metabolism were detected in rat tissues for the first time. These adducts were identified as the predominant exposed enal adducts and allowed reconstruction of complete enal metabolic pathways, revealing the rules of enal metabolism. Partial metabolic adducts derived from other enal types were also observed.

We summarized the dynamic changes of enal(C3:1), enal(C4:1), and enal(C5:1) and their adducts across multiple tissues in MI rats, with the results presented in Table 2. As shown in Fig. 5, two upstream metabolites in the enal–adduct metabolic pathway, free enals and GSH–enal adducts, exhibited parallel temporal trends in both heart and liver tissues following MI. In the heart, enal(C3:1), enal(C4:1), and enal(C5:1) gradually increased during the acute phase, peaked at 2 days post-injury, and then slowly declined during the tissue reparative and proliferative phase, returning to basal levels by day 14. In the liver, the corresponding GSH–enal adducts followed a similar pattern but with a delayed peak at 4 days post-injury. This temporal discrepancy indicates that MI-induced enals are transported via circulation to the liver, where they conjugate with GSH for detoxification and elimination, a critical pathway for the systemic clearance of lipid peroxidation-derived reactive aldehydes.

**Table 2:** Dynamic changes of enal(C3:1), enal(C4:1), enal(C5:1) and their adducts across multiple tissues in myocardial ischemia (MI) rats.

Compound	Tissue	Acute phase (2 h–2 d)	Tissue reparative and proliferative phase (4 d–14 d)
enal(C3:1)	Heart	Increase gradually and peak at 2 d	Decrease and return to basal level at 14 d
GSH–enal(C3:1)	Liver	Increase gradually	Peak at 4 d then decrease gradually
GlyCys–enal(C3:1)	Liver	Increase gradually and peak at 1 d	Gradually decrease
Cys–enal(C3:1)	Liver	Biphasic peaks at 2 h and 2 d	Gradually decrease
NAC–enal(C3:1)	Kidney	1st peak at 2 h	2nd peak after 14 d
enal(C4:1)	Heart	Increase gradually and peak at 2 d	Decrease and return to basal levels at 14 d
GSH–enal(C4:1)	Liver	Increase gradually	Peak at 4 d then decrease gradually
GlyCys–enal(C4:1)	Liver	Biphasic peaks at 2 h and 2 d	Gradually decrease
Cys–enal(C4:1)	Liver	1st peak at 12 h	2nd peak at 4 d then decrease gradually
NAC–enal(C4:1)	Kidney	1st peak at 12 h	2nd peak after 14 d
enal(C5:1)	Heart	Biphasic peaks at 12 h and 2 d	Decrease and return to basal levels at 14 d
GSH–enal(C5:1)	Liver	Increase gradually	Peak at 4 d then decrease gradually
GlyCys–enal(C5:1)	Heart	Fluctuate	Close to the basal level
Cys–enal(C5:1)	Liver	Fluctuate	Increase rapidly from 4 d and remain at a high level during 7–14 d
NAC–enal(C5:1)	Kidney	1st peak at 2 h	2nd peak at 4 d then decrease

GSH: glutathione; NAC: N-acetylcysteine.

**Fig. 5:**
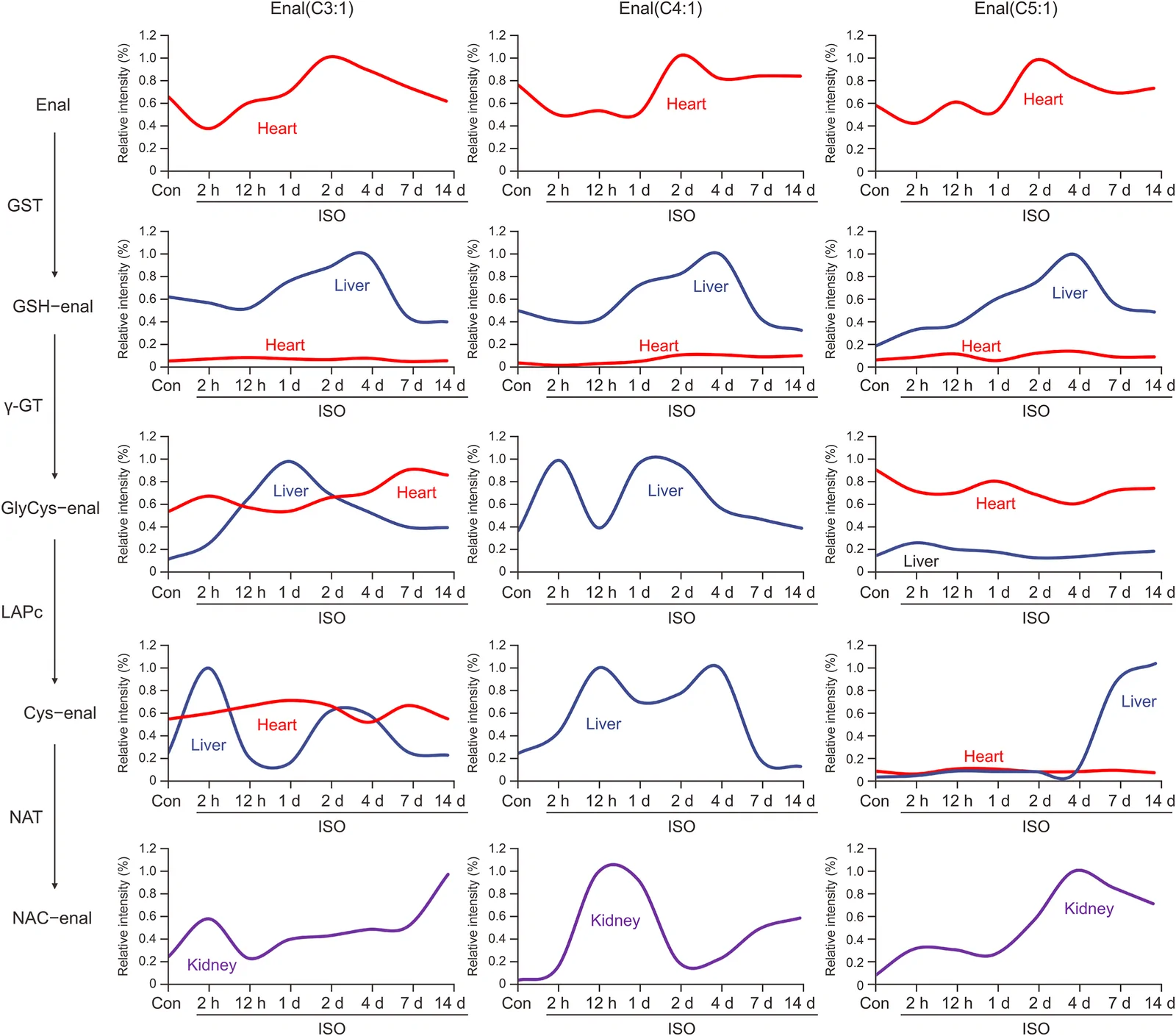
Dynamic changes of enal(C3:1), enal(C4:1), enal(C5:1), and their adducts across multiple tissues in myocardial ischemia (MI) rats. The horizontal axis represents time after MI, and the vertical axis represents the corresponding levels of enals and their adducts. GSH: glutathione; NAC: N-acetylcysteine; GST: glutathione S-transferase; γ-GT: γ-glutamyltransferase; LAPc: leucyl aminopeptidase; NAT: N-acetyltransferase; ISO: isoproterenol induced myocardial ischemia; Con: control.

Downstream metabolites of this pathway, including Cys–enal and NAC–enal adducts, also exhibited parallel temporal patterns in the liver and kidney. In the liver, Cys–enal(C3:1) and Cys–enal(C4:1) displayed biphasic profiles, with an initial rapid peak during the early acute phase (2–12 h post-injury), followed by a gradual decline, a second peak at the end of the acute phase (2–4 days post-injury), and subsequent slow reduction during the reparative and proliferative phase. In the kidneys, NAC–enal(C3:1), NAC–enal(C4:1), and NAC–enal(C5:1) also exhibited biphasic dynamics; however, the second peak of NAC–enal(C3:1) and NAC–enal(C4:1) occurred markedly later at 14 days post-injury, whereas the second peak of NAC–enal(C5:1) occurred at 4 days. These sequential kinetic differences highlight their metabolic hierarchy: Cys–enal adducts function as upstream intermediates, while NAC–enal adducts serve as downstream terminal metabolites.

GlyCys–enal adducts, occupying an intermediate stage in the *in vivo* enal–adduct metabolic pathway, exhibited complex temporal profiles. In the liver, GlyCys–enal(C3:1) mirrored the trend of upstream GSH–enal(C3:1), whereas GlyCys–enal(C4:1) aligned more closely with the dynamics of downstream Cys–enal(C4:1). This divergence reflects heterogeneity in enzymatic activities across different substrates. When upstream enzymatic activity predominates over downstream activity, GlyCys–enal profiles resemble those of upstream metabolites; conversely, they follow downstream metabolite dynamics when downstream activity dominates. If enzymatic activities at both stages are comparable or dynamically shift over time, GlyCys–enal levels may fluctuate, as observed for liver GlyCys–enal(C5:1). These patterns underscore the dynamic interplay of metabolic enzymes and their critical role in shaping the temporal landscape of enal–adduct metabolism.

Additionally, GSH–enal, GlyCys–enal, and Cys–enal adducts were detected in the heart, likely arising from two sources: (1) hepatic metabolism followed by circulatory transport back to the heart and (2) direct enal metabolism within cardiac tissue. Consequently, the temporal profiles of these enal adducts in the heart exhibited fluctuations, reflecting the combined influence of systemic redistribution and localized myocardial metabolic activity.

### 3.5 Dynamic profiles of enal adducts in heart, liver, and kidney of myocardial ischemic rats

Due to the tissue-specific distribution of enal–adduct metabolic enzymes, GST, γ-GT, and LAPc predominantly function in the liver [[14], [15], [16], [17]], whereas NAT primarily acts in the kidney [18]. Accordingly, this study focused on the target organ (heart) and metabolic organs (liver and kidney) of MI rats to characterize enal–adduct dynamics. Using the established analytical method, 10 GSH–enal, 11 GlyCys–enal, 4 Cys–enal, and 4 NAC–enal adducts were identified in the heart; 12 GSH–enal, 18 GlyCys–enal, and 9 Cys–enal adducts were identified in the liver; and 8 NAC–enal adducts were identified in the kidney. Cluster analysis was performed to categorize temporal profiles of enal adducts across these tissues. Time-dependent abundance patterns are presented as heatmaps and average time-course profiles in Fig. 6, with detailed data provided in Tables S14–S17.

**Fig. 6:**
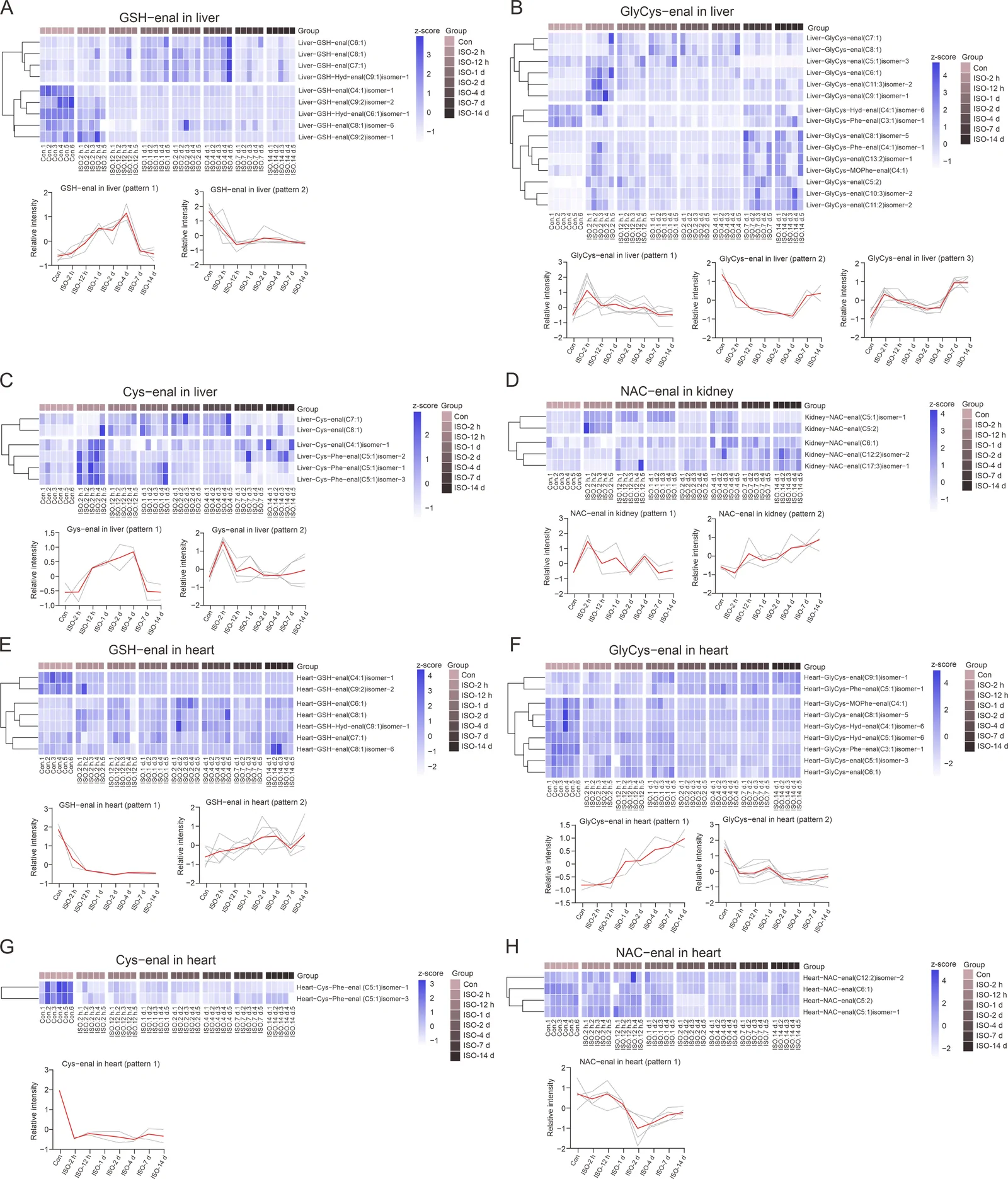
Heatmaps and average time-course profiles of enal–adducts in heart, liver, and kidney tissues of myocardial ischemia (MI) rats. (A) Glutathione (GSH)–enals in liver. (B) GlyCys–enals in liver. (C) Cys–enals in liver. (D) N-acetylcysteine (NAC)–enals in kidney. (E) GSH–enals in heart. (F) GlyCys–enals in heart. (G) Cys–enals in heart. (H) NAC–enals in heart. ISO: isoproterenol induced myocardial ischemia; Gly: glycine; Cys: cysteine; Con: control.

As shown in Figs. 6A–D, temporal profiles of enal–adducts revealed distinct metabolic dynamics in heart, liver, and kidney tissues. In the liver, GSH–enal adducts exhibited two patterns: (1) gradual accumulation during the acute phase, peaking at 1 day post-injury, followed by decline during tissue repair and proliferation, reflecting systemic detoxification via GSH conjugation; and (2) rapid depletion below baseline during the acute phase, likely due to competitive inhibition by pattern 1 adducts. GlyCys–enal adducts displayed three trends: (1) early acute-phase peaks with subsequent decline, (2) acute-phase depletion followed by recovery during the reparative phase, and (3) biphasic dynamics with dual peaks, highlighting variable enzymatic activity toward different substrates. Cys–enal adducts showed either progressive elevation followed by decline (pattern 1) or an early peak at 2 h post-injury with subsequent normalization (pattern 2). In the kidney, NAC–enal adducts exhibited fluctuating profiles throughout both phases, consistent with kidney excretion as a terminal clearance route.

The results indicate that during the acute phase of MI, levels of partial GSH–enal adducts, partial GlyCys–enal adducts, all Cys–enal adducts in the liver, and all NAC–enal adducts in the kidney peaked, suggesting that oxidative stress-induced enals are detoxified in the liver via GSH conjugation followed by stepwise metabolic processing. During the reparative and proliferative phase, the decline of upstream GSH–enal adducts and secondary peaks of select GlyCys–enal and Cys–enal adducts in the liver, along with sustained NAC–enal adducts in the kidney, indicate that the body completes free enal conjugation and prioritizes elimination of enal adducts through downstream metabolism.

Compared to the liver and kidney, the target organ heart exhibited fewer identified enal–adduct species and lower overall abundance. As shown in Figs. 6E–H, the temporal profiles of the four enal–adduct classes in heart tissues displayed fluctuating patterns. Nevertheless, a general trend emerged: during the acute phase of MI, most GSH–enal and GlyCys–enal adducts, along with select Cys–enal and NAC–enal species, reached peak concentrations. During the reparative and proliferative phase, levels of all enal–adducts declined to near-control values. These findings indicate that systemic detoxification via GSH conjugation and downstream metabolism effectively reduces the enal burden in the heart during tissue repair, reflecting the body's self-protective and reparative mechanisms against oxidative stress-induced damage.

Oxidative stress, defined as an imbalance between oxidant production and antioxidant defenses, is associated with a wide range of diseases [[7], [8], [9],[33], [34], [35]]. Oxidative stress-derived enals induce “carbonyl stress” [36] and form enal–protein adducts [6], which contribute to pathogenesis by depleting GSH [37], impairing protein function [38,39], disrupting lipid homeostasis [20], activating transient receptor potential ankyrin 1 (TRPA1) [[40], [41], [42], [43]], and triggering Ca^2+^ influx [42]. GSH binds enals to form GSH–enal adducts, which are subsequently metabolized and excreted via urine [[14], [15], [16], [17], [18], [19], [20]]. These metabolic and clearance pathways, as well as oxidative stress-induced damage, are consistent between humans and rats [2,28,44]. Because human tissues are generally unavailable, investigation in rat tissues provides a proxy for human enal metabolism. Although in this study enal adducts were only detected in rat tissues and not in rat or human blood and urine, the findings from the MI rat model still reflect relevant pathological processes in humans. Therefore, the analytical strategies established here for comprehensive characterization of GSH–enal adducts and their metabolites *in vivo* offer critical insight for the prevention and treatment of oxidative stress-related diseases.

While conjugation of enals with GSH represents the primary metabolic and detoxification pathway, other metabolic pathways have been less reported. For example, only 4-hydroxynonenal (Hyd–enal(C9:1)) and acrolein (enal(C3:1)) have been observed to undergo oxidation to carboxylic acids via aldehyde dehydrogenase (ALDH) [45,46], and Hyd–enal(C9:1) can be reduced to alcohols by alcohol dehydrogenase (ADH) [45]. The abundance of these redox metabolites is significantly lower than that of GSH-conjugated products [45]. Therefore, the redox metabolism of other enal species *in vivo* warrants further in-depth investigation using newly established analytical methods.

## 4 Conclusion

This study established a novel, comprehensive analytical strategy for characterizing GSH–enal adducts and their metabolites *in vivo*, based on DNPH derivatization coupled with LC-MS. This approach overcomes critical challenges in enal–adduct analysis, including poor stability, low sensitivity, and the lack of reference standards. To our knowledge, this is the first study to discover and identify 12 GSH–enal, 20 GlyCys–enal, 9 Cys–enal, and 8 NAC–enal adducts (encompassing three complete enal metabolic pathways) in rat heart, liver, and kidney, using a combination of precursor ion scanning, dMRM, HRMS, and a theoretical predicted database of 4496 enal–adduct candidates. Distinct temporal exposure profiles of enal adducts in MI rats were revealed: some exhibited dynamic changes correlated with myocardial injury and recovery processes, whereas others rapidly declined to baseline post-injury. Although human tissues were not available for this study, results from the MI rat models likely reflect relevant pathological processes in humans, given the conserved pathways for enal conjugation with GSH and subsequent metabolism. Therefore, the analytical strategy established here provides a powerful tool for investigating the pathogenesis and progression of oxidative stress-related diseases, as well as the mechanisms of action of therapeutic interventions.

## CRediT authorship contribution statement

**Jialong Jing:** Writing – review & editing, Writing – original draft, Methodology, Investigation, Data curation, Conceptualization. **Ning Sheng:** Supervision, Funding acquisition. **Zhe Wang:** Writing – review & editing, Validation, Supervision, Project administration, Investigation, Conceptualization. **Jinlan Zhang:** Writing – review & editing, Validation, Supervision, Project administration, Investigation, Funding acquisition.

## Declaration of competing interest

The authors declare that they have no known competing financial interests or personal relationships that could have appeared to influence the work reported in this paper.
