# Supplementary material for: Novel strategy for characterizing the metabolism of GSH–enal adducts in myocardial ischemia rat model using UHPLC–MS
Source: J Pharm Anal. 2026 Apr 17;16(10):101638. doi: 10.1016/j.jpha.2026.101638 (PMC13769228; doi:10.1016/j.jpha.2026.101638)
Supplement: Supplementary file 1 [file mmc1.docx]

**Supplementary Information**

**Novel strategy for characterizing the metabolism of GSH–enal adducts in myocardial ischemia rat model using UHPLC–MS**

**List of Supplementary information**

**Experimental details**

**Supplementary figures**

All supplementary figures can be found in this document.

**Figure S1.** Chemical structures of Enal standards.

**Figure S2.** Chemical structures of GSH–Enals.

**Figure S3.** Chemical structures of GlyCys–Enals.

**Figure S4.** Chemical structures of Cys–Enals.

**Figure S5.** Chemical structures of NAC–Enals.

**Figure S6**.Optimization of collision energy (CE) for Enal-adducts after DNPH (2,4-Dinitrophenylhydrazine) derivatization. MS/MS spectra of GSH–Enal(4:1)–DNPH at 10, 20, and 30 eV are shown as examples.

**Figure S7**.Optimization of precipitant for biological samples. (A) Derivatization and pre-treatment processes for biological samples. (B) EICs (Extracted Ion Chromatogram) of 3 representative types of Enal-adducts in rat liver prepared with different precipitant: acetonitrile, methanol, ethyl acetate, n-hexane, methanol (0.2%formic acid), acetonitrile (0.2%formic acid), MTBE-methanol (v/v,1/1), MTBE- acetonitrile (v/v,1/1). Extraction with MTBE-methanol (v/v, 1/1) yielded the highest response (1.4×10^4^) and the best peak shape.

**Figure S8.** HRMS/MS spectra of 15 GSH–Enal after DNPH derivatization via target MS/MS.

**Figure S9.** HRMS/MS spectra of 17 GlyCys–Enal after DNPH derivatization via target MS/MS.

**Figure S10.** HRMS/MS spectra of 11 Cys–Enal after DNPH derivatization via target MS/MS.

**Figure S11.** HRMS/MS spectra of 13 NAC–Enal after DNPH derivatization via target MS/MS.

**Figure S12.** The mass spectrometric fragmentation rules of GSH–Enal–DNPH

**Figure S13.** The mass spectrometric fragmentation rules of GlyCys–Enal–DNPH

**Figure S14.** The mass spectrometric fragmentation rules of Cys–Enal–DNPH

**Figure S15.** The mass spectrometric fragmentation rules of NAC–Enal–DNPH

**Figure S16.**TICs of GSH–Enal,GlyCys–Enal,Cys–Enal,NAC–Enal obtained by Precursor Ions.

**Figure S17.**Schematic diagram of the animal experimentation process.

**Figure S18.**Representative images of HE staining of rat heart tissues.

**Figure S19.**Physiological indicators in rats.(AST-plasma, SOD-heart).

**Supplementary tables**

All supplementary tables can be found in the additional sheets (Excel files).

**Table S1.** The parameters of target MS/MS scan mode for synthesized GSH–Enal–DNPH analysis

**Table S2.** The parameters of target MS/MS scan mode for synthesized GlyCys–Enal–DNPH analysis

**Table S3.** The parameters of target MS/MS scan mode for synthesized Cys–Enal–DNPH analysis

**Table S4.** The parameters of target MS/MS scan mode for synthesized NAC–Enal–DNPH analysis

**Table S5.** The standard curves, LOD, accuracy and precision of 12 Enal-adducts after derivatization

**Table S6.** The matrix effect and recovery of 12 Enal-adducts after derivatization

**Table S7.** Theoretical enal database containing 1124 enals

**Table S8.** Theoretical prediction Enal-adducts database containing 4496 Enal-adducts.

**Table S9.** The parameters of dMRM scan mode for GSH–Enal adducts candidates in rat organs

**Table S10.** The parameters of dMRM scan mode for GlyCys–Enal adducts candidates in rat organs

**Table S11.** The parameters of dMRM scan mode for Cys–Enal adducts candidates in rat organs

**Table S12.** The parameters of dMRM scan mode for NAC–Enal adducts candidates in rat organs

**Table S13.** The validated Enal-adducts and their dMRM scan mode parameters in rat organs

**Table S14.** Z-score normalized GSH–Enal detection results used for heatmap.

**Table S15.** Z-score normalized GlyCys–Enal detection results used for heatmap.

**Table S16.** Z-score normalized Cys–Enal detection results used for heatmap.

**Table S17.** Z-score normalized NAC–Enal detection results used for heatmap.

**1. Experimental details**

**1.1.Materials**

Enal standards (Fig S1) were purchased from J&K Scientific Ltd. (Beijing, China) and Anhui Zesheng Technology Co., Ltd. (Anhui, China). 2,4-Dinitrophenylhydrazine hydrochloride (DNPH) was obtained from Aladdin (Shanghai, China). GSH and Cys were purchased from J&K Scientific Ltd. (Beijing, China).GlyCys was purchased from Acmec Biochemical Technology Co., Ltd.(Shanghai, China). NAC was purchased from Aladdin Bio-Chem Technology Co., LTD.(Shanghai, China).

Ascorbic acid (AA) was purchased from J&K Scientific Ltd. (Beijing, China). Isoprenaline hydrochloride (ISO, 99%) was obtained from MedChemExpress (Shanghai, China). Ammonium acetate (NH_4_Ac, MS grade, ≥99.99%) were purchased from Sigma-Aldrich (St. Louis, MO, USA). Methanol (MeOH), acetonitrile (ACN), hexane, ethyl acetate and methyl tertiary butyl ether (MTBE) were purchased from Thermo Fisher Scientific (Morris Plains, NJ, USA). Formic acid (FA, HPLC grade, 99%) were purchased from Aladdin (Shanghai, China). Water was obtained from Wahaha Group Co., Ltd. (Hangzhou, China).

The heart marker enzymes AST (Aspartate Aminotransferase) plasma was determined using standard kits supplied from BioSino Bio-technology and Science, Inc. (Beijing, China). Antioxidant enzyme SOD (Superoxide Dismutase) was determined using standard kits purchased from Solarbio Science & Technology Co.,Ltd.(Beijing, China).The protein content of heart homogenates was determined with BCA protein assay kit purchased from Thermo Fisher Scientific (Morris Plains, NJ, USA).

**1.2.In vitro synthesis of Enal-Adducts**

A total of 50 μL aliquots of 17 enal solutions were precisely transferred into individual microcentrifuge tubes (EP tubes). These included 100 μmol/L six straight-chain enals (e.g., Enal(C3:1)), six branched enals (e.g., 3-Me-Enal(C4:1)), and five dienals (e.g., Enal(C6:2)). Subsequently, 50 μL of 10 mmol/L glutathione (GSH) in phosphate-buffered saline (PBS) was added to each tube. The mixtures were vortexed 10 min thoroughly and incubated in the dark at 37 °C for 3 hours to allow the formation of corresponding GSH-Enal adducts.

The preparation procedures for GlyCys-enal, Cys-enal, and NAC-enal adducts followed the same protocol under identical reaction conditions.

**1.3.LC-MS analysis conditions**

**1.3.1.** **UHPLC-Q-TOF/MS analysis**

In vitro synthesized Enal adducts were analyzed using an Agilent 1290 Series UHPLC system coupled with an Agilent 6550 Q-TOF mass spectrometer (Agilent Technologies, Santa Clara, CA, USA). A Waters (Milford, MA, USA) ACQUITY UPLC® BEH C18 column (1.7 μm, 2.1 × 100 mm, Waters Corporation) was used. The mobile phases were as follows: Phase A: water (containing 1 mmol/L ammonium acetate); Phase B: 95% acetonitrile (containing 1 mmol/L ammonium acetate). The gradient elution program was set as: 0–2 min, 10% B; 2–13 min, 10–100% B; 13–18 min, 100% B. The flow rate was 0.3 mL/min, column temperature was 30℃, and the injection volume was 10 μL.

The mass spectrometer operated in ESI negative ion target MS/MS mode. Ion source parameters were: sheath gas temperature 350°C, sheath gas flow rate 11 L/min, drying gas temperature 150℃, drying gas flow rate 14 L/min, nebulizer pressure 20 psi, capillary voltage 3500 V, nozzle voltage 500 V. Detailed Target MS/MS parameters are provided in Table S5–S8.

**1.3.2.** **UHPLC-MSQqQ/MS analysis**

Analysis of Enal adducts in biological samples was performed using an Agilent 1290 Series UHPLC system coupled with an Agilent 6470 QQQ mass spectrometer (Agilent Technologies, Santa Clara, CA, USA). A Waters (Milford, MA, USA) ACQUITY UPLC® BEH C18 column (1.7 μm, 2.1 × 100 mm) was used. The mobile phases were: Phase A: water (containing 1 mmol/L ammonium acetate); Phase B: 95% acetonitrile (containing 1 mmol/L ammonium acetate). The gradient elution program was: 0–2 min, 10% B; 2–40 min, 10–100% B; 40–45 min, 100% B. The flow rate was 0.3 mL/min, column temperature was 30℃, and the injection volume was 10 μL. Ion source parameters were identical to those set for the 6550 quadrupole time-of-flight mass spectrometer.

Precursor ion scanning mode was used to discover endogenous GSH-Enal, GlyCys-Enal, Cys-Enal, and NAC-Enal. The precursor ions were set as: m/z 306.1 for GSH-Enal-DNPH, m/z 177.0 for GlyCys-Enal-DNPH, m/z 120.0 for Cys-Enal-DNPH, and m/z 162.0 for NAC-Enal-DNPH.

dMRM scanning mode was applied to confirm endogenous GSH-Enal, GlyCys-Enal, Cys-Enal, and NAC-Enal. The confirmation ions were: m/z 272.1 for GSH-Enal-DNPH, [M-177-2H]⁻ for GlyCys-Enal-DNPH, [M-120-2H]⁻ for Cys-Enal-DNPH, and [M-162-2H]⁻ for NAC-Enal-DNPH.

The method for detecting Enal refers to our previous work^10^.

**1.4.Animal experiment**

Male Sprague-Dawley (SD) rats (250 g ± 20 g) were purchased from Spefoo Biotechnology Co., Ltd. (Beijing, China). All SD rats were housed under specific pathogen-free conditions: a 12-hour light/dark cycle, 23 ± 2℃ temperature, and (55 ± 5)% relative humidity. All rats were acclimated for one week before the start of experiments. All animal procedures were approved by the Animal Experiment Center of the Institute of Materia Medica, Chinese Academy of Medical Sciences & Peking Union Medical College (Ethics Approval No.: 00002254).

SD rats were randomly divided into two groups: Control group (Con, n=6) and myocardial ischemia model group (ISO, n=35). The ISO group was further subdivided into 7 subgroups based on sampling time points: ISO-2h, ISO-12h, ISO-1d, ISO-2d, ISO-4d, ISO-7d, and ISO-14d (n=5 per subgroup). Myocardial ischemia was induced by intraperitoneal injection of isoproterenol (ISO, 5 mg/kg/day) once daily for 3 consecutive days^22^. Rats in the Con group received an equal volume of normal saline. At 2 h, 12 h, 1 d, 2 d, 4 d, 7 d, and 14 d after modeling, rats were euthanized by CO_2_ inhalation, and samples were collected. Whole blood was collected into heparin sodium-containing tubes, centrifuged at 4000 rpm to obtain plasma, and stored at -80℃. Heart, liver, and kidney tissues were excised, minced into small pieces, and homogenized with normal saline containing 0.5% ascorbic acid (AA)^10^ at a mass-to-volume ratio of 1:4 (w/v). Tissue homogenates were stored at -80℃. Collected heart, liver, and kidney tissues were used for Enal-adduct analysis, heart HE staining, and biochemical index analysis.

**1.5.Histopathological section**

Immerse the dissected and clean cardiac tissue in 4% formalin solution for fixation of cardiac tissue for 1 day. Embed the tissue in paraffin, which provides support and allows for thin sectioning for the histopathological examination(HE staining).

**1.6.Determination of biochemical indices**

The heart marker enzymes AST plasma was determined using standard kits supplied from BioSino Bio-technology and Science, Inc. (Beijing, China).Antioxidant enzyme SOD was determined using standard kits purchased from Solarbio Science & Technology Co.,Ltd.(Beijing, China).

**2. supplementary figures**

Figure S1. Chemical structures of 17 Enal standards.

**Figure S2.** Chemical structures of 15 synthesized GSH–Enal adducts.

**Figure S3.** Chemical structures of 17 synthesized GlyCys–Enal adducts.

**Figure S4.** Chemical structures of 11 synthesized Cys–Enal adducts.

**Figure S5.** Chemical structures of 13 synthesized NAC–Enal adducts.

**Figure S6**.Optimization of collision energy (CE) for Enal-adducts after DNPH (2,4-Dinitrophenylhydrazine) derivatization. MS/MS spectra of GSH–Enal(4:1)–DNPH at 10, 20, and 30 eV are shown as examples.

**Figure S7**.Optimization of precipitants for biological samples. (A) Derivatization and pre-treatment processes for biological samples. (B) EICs (Extracted Ion Chromatogram) of 3 representative types of Enal-adducts in rat liver prepared with different precipitant: acetonitrile, methanol, ethyl acetate, n-hexane, methanol (0.2%formic acid), acetonitrile (0.2%formic acid), MTBE-methanol (v/v,1/1), MTBE- acetonitrile (v/v,1/1). Extraction with MTBE-methanol (v/v, 1/1) yielded the highest response (1.4×10^4^) and the best peak shape.

**Figure S8.** HRMS/MS spectra of 15 synthesized GSH–Enal adducts after DNPH derivatization.

**Figure S9.** HRMS/MS spectra of 17 synthesized GlyCys–Enal adducts after DNPH derivatization.

**Figure S10.** HRMS/MS spectra of 11 synthesized Cys–Enal adducts after DNPH derivatization.

**Figure S11.** HRMS/MS spectra of 13 synthesized NAC–Enal adducts after DNPH derivatization.

**Figure S12.** The mass spectrometric fragmentation rules of GSH–Enal–DNPH

**Figure S13.** The mass spectrometric fragmentation rules of GlyCys–Enal–DNPH

**Figure S14.** The mass spectrometric fragmentation rules of Cys–Enal–DNPH

**Figure S15.** The mass spectrometric fragmentation rules of NAC–Enal–DNPH

**Figure S16.**TICs of GSH–Enal, GlyCys–Enal, Cys–Enal, and NAC–Enal adducts obtained by precursor ion scanning.

**Figure S17.**Schematic diagram of the animal experimentation process.

**Figure S18.**Representative images of HE staining of rat heart tissues.

**Figure S19.** Biochemical indicators in rats.(AST-plasma, SOD-heart).
